# Grain color formation and analysis of correlated genes by metabolome and transcriptome in different wheat lines at maturity

**DOI:** 10.3389/fnut.2023.1112497

**Published:** 2023-02-07

**Authors:** Li Li, Hui Zhang, Junna Liu, Tingzhi Huang, Xuesong Zhang, Heng Xie, Yirui Guo, Qianchao Wang, Ping Zhang, Peng Qin

**Affiliations:** College of Agronomy and Biotechnology, Yunnan Agricultural University, Kunming, China

**Keywords:** colored wheat, maturity, grain color, metabolome, transcriptome

## Abstract

Colored wheat has been recognized broadly for its nutritional value because of its natural content of the colorant anthocyanin. To investigate the reasons for the formation of the wheat grain color at maturity, metabolomic and transcriptomic analyses were performed on three different grain colors of wheat. Through metabolome analysis, 628 metabolites were identified. Of the 102 flavonoids, there are 9 kinds of anthocyanins related to color formation, mainly cyanidin and peonidin, and their metabolite content was the lowest in white-grain wheat. Among the genes associated with color formation, the structural gene TraesCS2D02G392900 in F3H with the bHLH transcription factor could elucidate the origin of wheat coloration. Multi-omics analysis showed that color formation is mainly influenced by the regulation of genes affecting anthocyanin and related synthesis. The results of this study may provide a theoretical basis for grain color formation at maturity and the nutritional and product development potential of colored wheat lines.

## Graphical Abstract

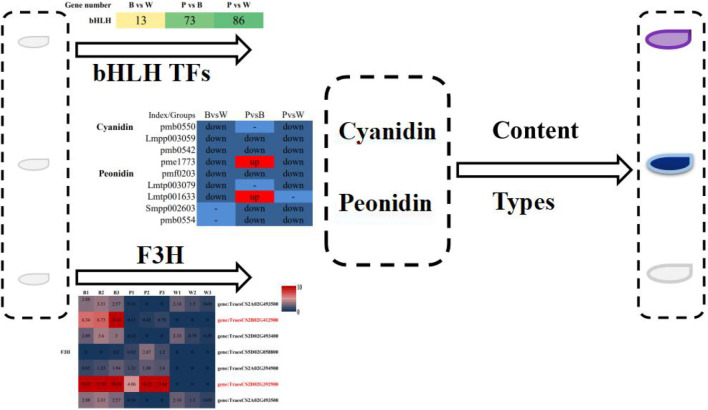

## 1. Introduction

Cereals are the primary source of food and feed worldwide and contribute to more than half of the calories consumed by humans (1). Nowadays, consumer needs have changed from a diet that provides energy to one that is nutritionally balanced and functionally healthy. Wheat (*Triticum aestivum* L.) is one of the major cereals, and its starch, protein, mineral, and dietary fiber content are the primary sources of nutritional value for wheat consumers. Colored wheat merits extensive research due to its nutritional and antioxidant properties, as well as its natural colorants (2). It is a source of essential nutrients and an array of bioactive compounds, such as anthocyanins, carotenes, and phenolic acids. These compounds are known for their high antioxidant activity (3). Colored wheat, for example, contains anthocyanins, which have a high antioxidant capacity and may help prevent chronic diseases such as obesity, cancer, and cardiovascular disease, as well as delay aging (4). The presence of carotenoids and catechol in the outer layer of white-grain wheat results in a red color with a hint of yellow. Unlike common wheat, the color of colored wheat is mainly related to anthocyanins. Colored wheat is also rich in tocopherols, phenolic acids (5, 6), and trace elements required for normal human metabolism (7), with the main tocopherols being α-tocopherol and β-tocotrienol (5, 6). Reducing synthetic food colorants and dyes is a significant goal in food research and industry. Colored wheat, having natural pigments, can be used as an alternative food colorant in the food processing industry. Furthermore, as a functional food, it enhances the nutritional value and sensory properties of food (2). Therefore, colored wheat presents several health benefits for consumers, which is a significant objective of research in wheat breeding and baking. The anthocyanin coloration of the pericarp and, or aleurone was proposed as a marker for wheat lines with unique nutritional qualities. Most colored wheat lines are characterized by poor resistance and low yields. In order to choose and sustainably breed high-anthocyanin lines, it is crucial to comprehend the biosynthesis routes of anthocyanins in various colored grains as well as the regulatory mechanisms that control their expression.

Previous studies on grain color have been carried out at the metabolite and genetic levels. The differences in grain color were caused by the presence of polyphenols, tannins, anthocyanins, and carotenoids; blue aleurone (*Ba* genes), purple pericarp (*Pp* genes), and yellow endosperm (*Psy* genes) were determined by the presence of anthocyanins and carotenoids, respectively (8). Phenolic acids and flavonoids are the main phenolic compounds found in wheat. Despite their low concentrations, they are the primary causes of wheat pigmentation and have a significant impact on grain quality (9). While white (red) is the most common color of wheat grains, other colors, such as blue and purple, are also documented. This color difference is mainly due to the flavonoid content in the aleurone layer and seed coat of wheat grains (10). Genetically, red grains are controlled by 1–3 dominant alleles *R-A1* (on chromosome 3AL), *R-B1* (3BL), and *R-D1* (3DL), and white-grains are determined by the recessive alleles *r-A1, r-B1*, and *r-D1* (11). The color of purple grains is caused by the purple pericarp *Pp* gene, and the *Pp1, Pp3b*, and *Pp3* genes have been identified (12). Blue grain color is determined by genes for blue aleurone *Ba* (13). The yellow endosperm color is determined by two loci *Psy1* and *Psy2*, located on homoeologous chromosome groups 7 and 5 (14). Considering the metabolites, red and white-grains colors are composed of catechins and tannin derivatives (11). Purple grain pigments, on the other hand, are due to the presence of anthocyanins in the grain’s surface layer (pericarp) (12). In blue aleurone wheat, the main anthocyanins were delphinidin 3-glucoside and delphinidin 3-rutinoside (13). The yellow coloration of the endosperm is associated with the presence of carotenoids (14). The biosynthesis of anthocyanins in wheat grains results in the formation of blue, purple, or a combination of the two colors (15): proanthocyanidins in the wheat seed coat results in reddish-brown color (16), biosynthesis of anthocyanins in the aleurone layer results in blue-gray color, and biosynthesis of anthocyanins in pericarp cells results in purple color (17).

Most studies have shown that colored wheat has a higher nutritional value than regular wheat. The improvement in the composition of radiation-induced mutants in purple wheat was evaluated using nuclear magnetic resonance spectroscopy. The study identified 33 metabolites where the content of primary metabolites in colored wheat is higher than that in yellow wheat (18). The diversity of bioactive compounds in colored wheat grains was studied using high-performance liquid chromatography-mass spectrometry (HPLC-MS/MS) and was found to be considerably higher than that in white-grain wheat. The anthocyanins in colored wheat flour have a stronger antioxidant capacity and greater antibacterial potential than those in white-grain wheat, and therefore, are natural plant-based antibacterial agents (19). The anti-inflammatory effect of purple wheat extract was the strongest in white-grain wheat and colored wheat, while white wheat had the weakest, and the contents of iron and zinc in colored wheat lines were higher, indicating that colored wheat has double biological enhancement (20). The phenolic acid and flavonoid species in purple, yellow, and white wheat grains were similar, but their anthocyanin contents differed. Purple wheat has a high concentration of anthocyanins and a high antioxidant capacity (9). Animal experiments were conducted to study the anti-aging and antioxidant properties of anthocyanin-rich purple wheat. In experiments where colored wheat was added to the animal diet, the purple wheat diet restored glucose and insulin sensitivity in the mice, thereby preventing obesity and related complications (21). Anthocyanins from purple wheat increased the stress response of *Caenorhabditis elegans*, reduced oxidative stress, significantly extended the average cell life span, and exhibited anti-aging effects (22). They may exert similar properties in humans, which is a question to be explored in future studies. The genes that control anthocyanin biosynthesis are considered for wheat breeding programs intended to improve the nutritional value of whole-grain products (1, 23). Thus, biochemical and *in vitro* cell line research showed that colored wheat has an absolute advantage over white-grain wheat because of its unique nutritional quality.

The formation of the grain color of colored wheat is a dynamic process, which is affected not only by environmental factors but also by internal factors such as bioactive substances, structural genes, and regulatory genes (24). In this study, the metabolites of wheat grains of three colors (white, blue, and purple) were identified using HPLC-MS/MS. The transcriptome and metabolome were integrated to investigate the mechanism of kernel color formation during maturation of three different colored wheat grains. Finally, differential metabolites and differential genes revealed the metabolic pathways and candidate genes for wheat grain coloration. This experiment provides new insights on the color formation of wheat seeds at the seed filling and maturity stages, which will help make full use of colored wheat lines.

## 2. Materials and methods

### 2.1. Overview of plant materials

**FIGURE 1:**
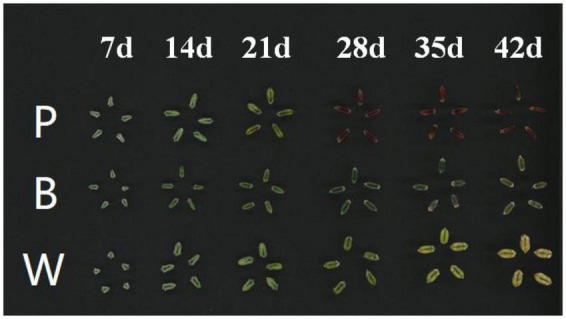
Sampling seed map of wheat after flowering. The figure was obtained using a camera.

Advanced generation strains of wheat (Dianmai 20-1, Dianmai 20-8, and Dianmai 16) were selected in-house from which grains comprising the colors purple (P), blue (B), and white (W) were used as materials for the experiment (Figure 1). Dian20-1, Dian 20-8, and Dianmai 16 are near-isogenic lines belonging to the same genetic background. Among them, Dian 20-1 is an advanced generation line (F7) that is the result of crossing Dianmai 16 with Pibian 05G68, whereas Dian 20-8 is an advanced generation line (F7) that is the result of crossing Dianmai 16 with AS905001. Barring the different material lines, the field cultivation and management conditions remained the same. The samples of this experiment were all wheat seeds at the same developmental stage of dough stage (42 days after flowering). Maturity was determined by observing the morphology of the wheat plant and the grain morphology. When the inner and outer glumes of the florets in the middle of the spike open, the anthers are scattered when the first day of flowering is marked by a tag. 42 days after flowering more than 50% of the wheat plants in the field wheat wax ripening stage seed discoloration (white-grain wheat becomes yellow, blue wheat becomes dark blue, purple wheat becomes dark purple), the endosperm of the grain is waxy, no pulp in the grain, hardness with the ripening process from soft to hard, wheat grain can be broken by nails, moisture content between 25 and 35%. Wax ripening period leaves yellow but not dry, wheat stalks become yellow, only slightly green on the stem nodes. The material was planted in the open on flat terrain with easy access to drainage and irrigation. The land was loosened and fertilized before planting, and weeding was performed during the wheat seedling stage in November 2019. The same-day flowering spikelets were marked in three wheat lines, and six spikelets from the middle of the wheat spike were sampled regularly 1 week after anthesis until maturation (7–42 days). Fresh samples taken from the plants were rapidly treated with fluid nitrogen and then transferred to a fridge at −80°C until utilized next. Mature wheat (42 days after flowering) was chosen for metabolite assurance and transcriptome sequencing, where the transcriptome data were approved by real-time quantitative PCR (RT-qPCR, Foster City, CA, USA). Each experiment was conducted in triplicates.

### 2.2. Metabolomics determination and analysis

The wheat seeds were ground in a blender (MM 400, Retsch, Haan, Deutschland) for 1.5 min, extricated with 70% methanol, and stored at 4°C after vacuum freeze-drying (Scientz-100F, Ningbo, China). The suspension was centrifuged (rotation speed 12,000 rpm, 10 min) (ANPEL, Shanghai, China),^1^ and the supernatant was sifted for the procurement and investigation of UPLC-MS/MS (UPLC, SHIMADZU Nexera X2,^2^ MS, Applied Biosystems 4500 Q TRAP)2, information. Sample extract chromatography-mass spectrometry data were collected using UPLC-MS/MS. An Agilent SB-C18 column (1.8 μm, 2.1 mm × 100 mm) (California, USA) was set to 40°C for chromatographic separation, and acetonitrile and pure water were added with 0.1% formic acid to form the mobile phase. The mass spectrometry conditions of electrospray ionization (ESI) temperature, voltage, curtain gas (CUR), and collision-activated dissociation (CAD) parameters were set to 550°C, 5,500 V, 30 psi, and high, respectively. The metabolite identification annotation was based on the self-built metware database (MWDB)^3^ of Wuhan Metaville Biotechnology Co., and the substance characterization was based on secondary spectrum information. Metabolite characterization and quantification were performed in a multiple reaction monitoring (MRM) mode based on secondary spectral information and triple quadrupole mass spectrometry, respectively. The mass spectral peaks of each metabolite identified within the nine samples are redressed to guarantee the exactness of the subjective and quantitative investigation of the metabolites. Quality control (QC) samples were used to determine the technical reproducibility of metabolite extraction and detection during the instrumental analysis. All the identified metabolites were analyzed for overall metabolic variation, between lines and between different biological replicates of the same variety using principal component analysis (PCA) and orthogonal partial least squares discriminant analysis (OPLS-DA). Based on the OPLS-DA results, those with Variable Importance in Projection (VIP) ≥ 1 and | fold change| ≥ 2 or | fold change| ≤ 0.5 were selected as differential metabolites.

### 2.3. Transcriptome sequencing

Triplicates of the three lines were prepared for transcriptome sequencing. RNA extraction and detection in samples were optimized and improved based on a previous study (25). RNA integrity and the presence of DNA contamination were verified by agarose gel electrophoresis. The Agilent 2100 Bioanalyzer (California, USA) and Qubit 2.0 fluorometer (Massachusetts, USA) further accurately detected the RNA integrity and concentration. The mRNA obtained from the samples is synthesized into double-stranded cDNA by reverse transcription. After treatment and purification of the double-stranded cDNA, the cDNA library was enriched by PCR. The initial quantification and detection of the insert size of the library were then completed using Qubit 2.0 and Agilent 2100. After quality verification, the different libraries were sequenced using the Illumina HiSeq platform. The clean reads used for analysis were checked for the sequencing error rate and GC content distribution, and data filtering removed the reads with adapters. When the *N* content in any sequencing read exceeds 10% of the number of bases in that read, or when the number of low quality (*Q* < = 20, the number of bases with Qphred values not less than 20 as a percentage of the total number of bases) bases contained in any sequencing read exceeds 50% of the number of bases in that read, both remove the paired reads to obtain high quality reads. The raw RNA sequences were quality-controlled, and low-quality reads were subsequently removed. The processed reads were mapped by sequence alignment with the reference genome. Fragments per kilobase of transcript per million fragments mapped (FPKM) was used to measure of transcript or gene expression levels. This experiment had three biological replicates, where the investigation of differential expression between test bunches was performed utilizing DESeq2 (26, 27). Differential genes required not only | log_2_ fold change| ≥ 1, but also the false discovery rate (FDR) < 0.05.

### 2.4. Transcriptome data analysis

After screening for differential genes, the genes were expressed in different sample groups for differential expression analysis. Their expression level was analyzed using differentially expressed genes (DEGs) functional annotation and functional enrichment, variable splicing analysis, novel gene discovery, and annotation-focused analysis. The Kyoto Encyclopedia of Genes and Genomes (KEGG) annotation focused analysis was performed on DEGs, in addition to gene ontology (GO), Karyotic Orthologous Groups (KOG), Pfam, Swiss-Prot, TrEMBL, and NR databases for functional annotation. Differential gene annotation using the KEGG database and GO provided a systematic analysis of big trial data. The protein sequences or cDNA sequences were aligned to the KOG (28) database using the Blast software, after which the annotations from the KOG database were extracted. The transcription factors (TF) were identified by hmmscan matching using the defined TF families and rules in the database. Based on the location information of the reads of the genome on the comparison, the reads were assembled into transcripts using StringTie and then compared with the annotation information of the genome using GffCompare to find new transcripts or new genes. The alternative splicing (AS) analysis of transcriptome data was implemented using rMATS. The rMATs are quantified by JC and JCEC, with JC meaning that only reads across the splicing join point are used for quantification, while JCEC means reads across the splicing join point. The reliability of transcriptome sequencing results was verified by RT-qPCR for all lines, with the ATP-dependent 26S proteasomal regulatory subunit (26S) serving as an internal reference. The gene primers (Supplementary Table 1) were designed using Beacon Designer 7.9. The DEGs were randomly selected for the RT-qPCR assay with of each variety. The relative expression levels of genes were calculated using the 2^–ΔΔCt^ method (29).

### 2.5. Integration analysis of metabolomics and transcriptomics

The causes of wheat color formation were analyzed based on the results of differential metabolites combined with the results of transcriptomic differential genes. The genes and metabolites detected in each differential grouping were subject to Pearson correlation analysis. The results with correlation coefficients greater than 0.8 were selected for correlation coefficient clustering heat map and correlation network diagram analysis. The two-way orthogonal partial least squares (O2PLS) model was employed to integrate and evaluate the overall correlation of the metabolomic and transcriptomic data.

## 3. Results

### 3.1. Qualitative and quantitative analysis of metabolites in wheat seeds among different lines

**FIGURE 2:**
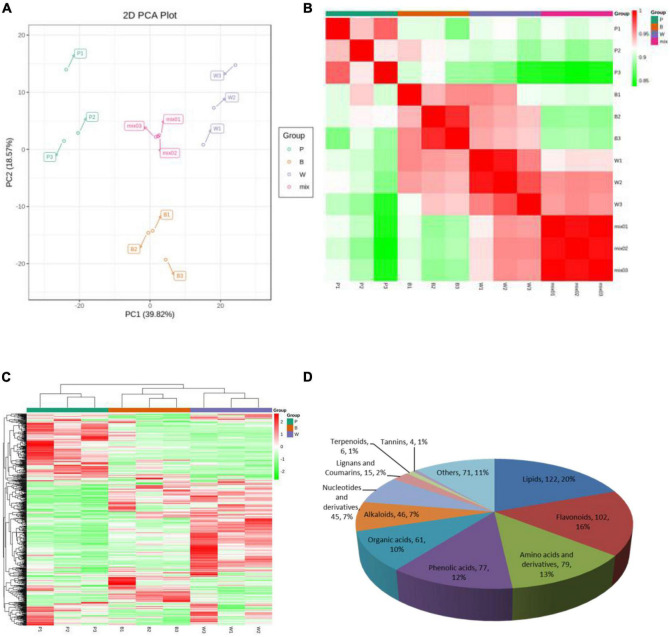
Mass spectrometry data of quality control wheat samples. **(A)** Principal component analysis (PCA) score map, where “mix” represents a mix of three lines. **(B)** Correlation map among samples. The horizontal coordinate of the correlation graph between samples indicates the sample name, the vertical coordinate indicates the corresponding sample name, and the color represents the correlation coefficient value. **(C)** Overall cluster diagram of samples. In the overall clustering plot, the sample name is presented in the horizontal coordinate, the metabolite information is presented in the vertical coordinate, where “Group” represents each group, “Class” represents the substance classification, and red represents high content, green represents low content. **(D)** Classification map of 628 metabolites.

Metabolite quantification was accomplished using MRM (Supplementary Figures 1A, B). The high overlap of the curves was demonstrated by overlapping the total ion flow plots detected by mass spectrometry of different QC samples, indicating good instrument stability, technical reproducibility, and data reliability (Supplementary Figures 1C, D). The PCA score plot (Figure 2A), the correlation plot between samples (Figure 2B), and the cluster analysis heat map (Figure 2C) demonstrated good biological repeatability of the experiment. Here, 628 metabolites were identified in P, B, and W lines and consisted of 11 classes. Lipids, flavonoids, amino acids, and their derivatives were the three major classes with 122, 102, and 79 metabolites, respectively (Figure 2D), among which flavonoids were associated with the color formation of wheat grains.

### 3.2. Analysis of metabolite differences between different lines of wheat seeds

**FIGURE 3:**
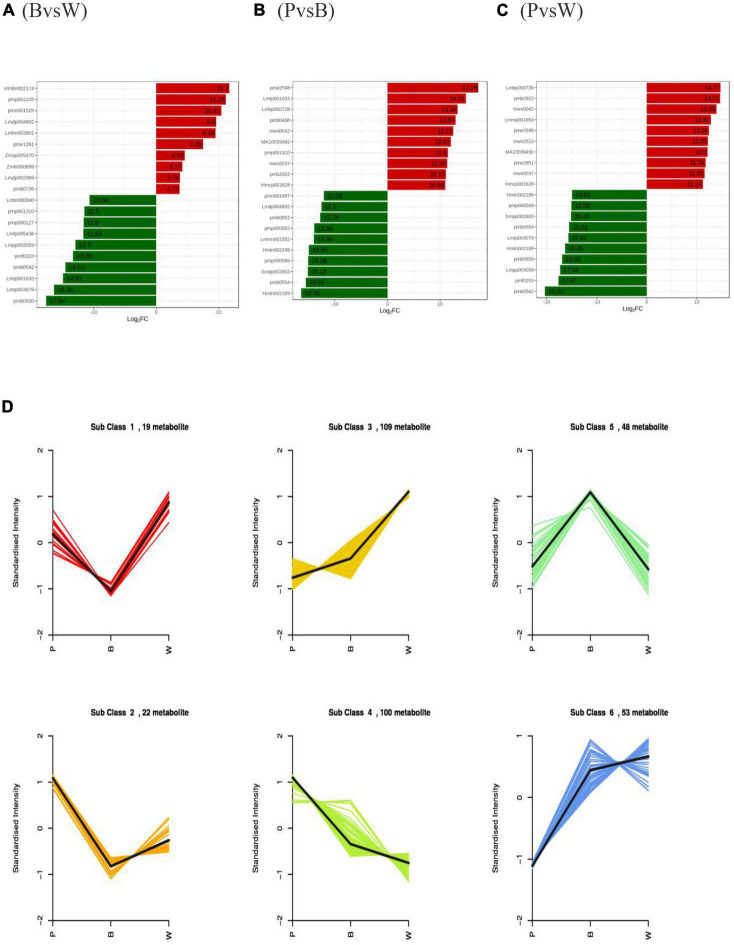
Differential metabolites profiles **(A)** Multiple histograms for blue and white wheat grains. **(B)** Multiple histograms for purple and blue wheat grains. **(C)** Multiple histograms for purple and white wheat grains. **(D)** K-means clustering. Metabolites with the same relative content variation trend in different lines were classified into one subclass.

The differences in wheat seeds were initially verified by PCA, which showed a clear trend of separation between groups, differences between groups, and good reproducibility of samples within groups (Supplementary Figures 2A–C). All three sets of the OPLS-DA validation plots (Supplementary Figures 2D–F) have shown that the R2Y and Q2 values were > 0.9 (*p* < 0.05), indicating that the proposed model was stable, reliable, and appropriate for this experiment. The differential metabolites among the different lines were screened based on the results of OPLS-DA combined with the screening criteria of differential metabolites used in this study. The samples were grouped as blue and white (BvsW), purple and blue (PvsB), and purple and white (PvsW) to compare the differential metabolites. There were 157 (92 upregulated and 65 downregulated), 221 (116 upregulated and 105 downregulated), and 263 (155 upregulated and 108 downregulated) differential metabolites in the BvsW, PvsB, and PvsW groups, respectively (Supplementary Table 2). The VIP values of differential metabolites (Supplementary Figures 2G–I) and differential multiplicity histogram (Figures 3A–C) and revealed that the differential metabolites with large variations among the three groups were flavonoids, amino acids and their derivatives, and phenolic acids. In particular, the top ten metabolites downregulated in the PvsW group were all flavonoids. K-means cluster analysis of differential metabolites was carried out to understand the trend of the relative content of metabolites in the three wheat grain colors. It was found that the 351 substances comprised 83 flavonoids, 53 phenolic acids, 43 amino acids and their derivatives, and 43 lipids (Figure 3D and Supplementary Table 3). The flavonoids and phenolic acids were more dominant in Subclass 3, 4, and 5. Thirteen flavonoids and 16 phenolic acids showed the same trend of relative content change in Subclass 3, and the highest relative content in sample W; 38 flavonoids and 13 phenolic acids showed the same trend of relative content change in Subclass 4, and the highest relative content in sample P; 17 flavonoids and 13 phenolic acids showed the same trend of relative content change in Subclass 4, and the highest relative content in sample P. The relative contents of 17 flavonoids and 11 phenolic acids showed the same trend in Subclass 5, and the relative content of sample B was the highest. The relationship between the differential metabolites of each group is also shown in the form of a Wayne diagram, and 56 distinctive metabolites were noted among the three comparison bunches, with 28, 35, and 54 differential metabolites specific to the BvsW, PvsB, and PvsW groups, respectively (Supplementary Figure 3). Different metabolites interact in the organism to form different biosynthetic pathways. The KEGG classification map of differential metabolites showed that the three differential groups were followed by the amino acid biosynthesis pathway (Supplementary Figures 4A–C), except for the primary and secondary metabolic pathways, which accounted for a larger proportion of the differential metabolites. The differential metabolite KEGG enrichment plots demonstrated significant enrichment in the anthocyanin biosynthesis pathway (Supplementary Figures 4D–F). In the anthocyanin biosynthesis pathway, nine anthocyanins were identified, namely, kuromanin, keracyanin, cyanidin-3-*O-*(6″-*O-*acetyl) glucoside, cyanidin-3-*O-*(6″-*O-*malonyl) glucoside, peonidin-3-*O-*glucoside, peonidin-3-*O-*(6″-*O-*acetyl) glucoside, peonidin-3-*O-*rutinoside, pelargonidin-3-*O-*(6″-*O-*malonyl) gluco-side, and peonidin-3-*O-*rutinoside-5-*O-*glucoside.

### 3.3. Transcriptome analysis of different wheat grains

**FIGURE 4:**
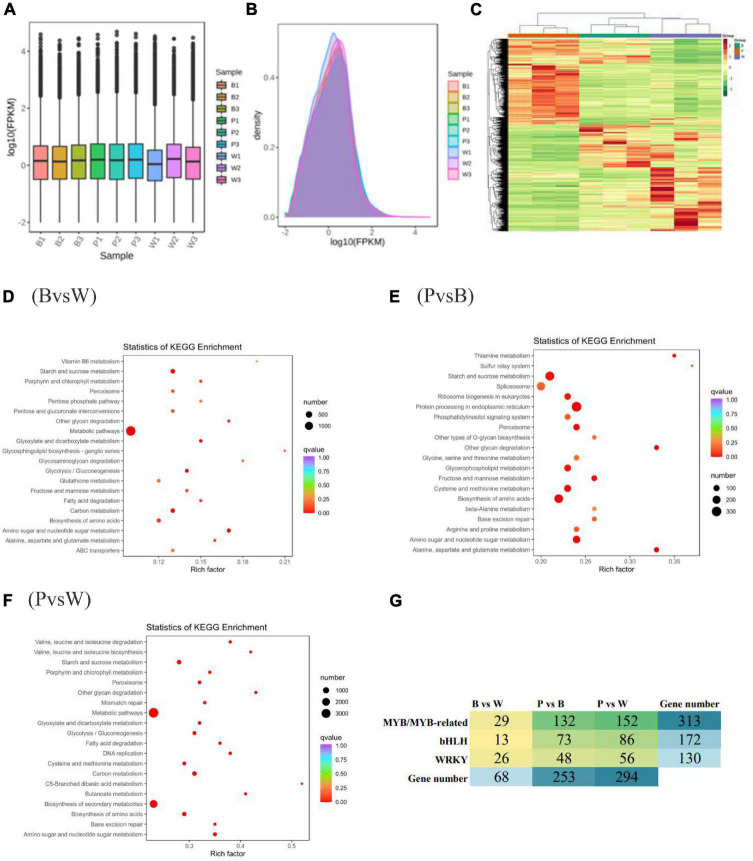
**(A)** Box-and-whisker plot of gene expression level. **(B)** Gene expression density distribution map. **(C)** Heat map of differential gene clustering. The horizontal and vertical coordinates indicate the sample name and stratified clustering results for differential genes. **(D–F)** Scatter plot of differential gene KEGG enrichment. The horizontal and vertical coordinates represent the enrichment factor and KEGG pathway, respectively. The larger the enrichment factor, the redder it is, indicating the more enrichment quantity and high enrichment degree. **(G)** Heat map showing the number of different transcription factors (TF) families in different comparison groups.

In this experiment, the transcriptomes of three different grain color wheat seeds (three biological repeats of each variety) were sequenced and analyzed, and an additional 61.95 GB of clean data were obtained. The clean data of each sample amounted to 6 GB with ≥92% Q30 bases (Supplementary Table 4). The clean reads after QC were compared to the reference genome and presented comparison efficiencies of >90%, much higher than 70%. This shows that the sequencing data met the requirements of subsequent analysis. FPKM was adopted as a gene expression level measurement so that the fragment number truly reflects the expression level of transcripts. The expression box line plot (Figure 4A) and density distribution plot (Figure 4B) reflected the minimal difference in the overall gene expression levels of different samples, and most genes had log10 (FPKM) values between −2 and 2. The combined correlation heat map and PCA score plot showed that the samples were biologically reproducible and different lines of wheat clustered separately (Supplementary Figures 5A, B). In summary, it was prepared to further search for DEGs. The genes detected in this experiment were functionally annotated with KEGG, GO, KOG, Pfam, Swiss-Prot, TrEMBL, and NR, were 76,228; 83,510; 93,319; 246,558; 75,389; 107,372, and 106,835 genes were annotated, respectively.

### 3.4. Differential gene analysis of different wheat grain transcriptomes

The differential genes were obtained according to the screening criteria for differential genes specified in the method. The analysis of DEGs was completed using DESeq2. The comparison of BvsW, PvsB, and PvsW showed the presence of 8357 (4,400 upregulated, 3,957 downregulated), 17,427 (9,111 upregulated, 8,316 downregulated), and 21,703 (11,941 upregulated, 9,762 downregulated) genes, respectively (Supplementary Table 5). The overall distribution of gene expression levels and differential ploidy can be visualized by MA plots (Supplementary Figures 5C–E). To investigate the expression patterns of genes in wheat with different seed colors, k-means clustering analysis of FPKM of genes was conducted. The same class of genes had similar change trends under different experimental treatments and might have similar functions. Subclass 6, Subclass 4, and Subclass 5 showed opposite expression trends, which could be used as potential markers to distinguish different seed colors (Supplementary Figure 5F). After extracting the centralized and normalized FPKM expressions of differential genes and performing hierarchical clustering analysis of the clustering heat map, it was observed that the DEGs with high expression levels clustered differently in each wheat group (Figure 4C). Different gene products in organisms perform various biological functions through interactions. The DEG pathways were annotated with the highest metabolic DEGs of all five categories. The number of DEGs annotated in each KEGG pathway is presented in the KEGG classification bar chart and shows that the metabolic pathways and secondary metabolic pathways are more predominant (Supplementary Figures 6A–C). The scatter plots of differential gene KEGG enrichment showed that starch and sucrose metabolism, amino sugar and nucleotide sugar metabolism, biosynthesis of amino acids, and peroxisome were significantly enriched in all groups (Figures 4D–F). The enrichment analysis of DEGs by GO classification showed that among the three components of molecular function, biological process, and cellular component, the biological process was more predominant in the three groups (Supplementary Figures 6D–F). The top three functional categories of DEGs analyzed by KOG annotation were general function prediction only and post-translational modification, protein turnover, chaperones, and signal transduction mechanisms (Supplementary Figure 7).

The number of the basic helix-loop-helix (bHLH) transcription factors (TFs) associated with wheat color formation were 13, 73, and 86 in BvsW, PvsB, and PvsW, respectively. There were 29 MYB and MYB-related TFs in BvsW, 132 in PvsB, and 152 in PvsW. Similarly, there were 26 WRKY-related TFs in 26 in BvsW, 48 in PvsB, and 56 in PvsW (Figure 4G). MYB expression was mostly upregulated in BvsW, PvsB, and PvsW, and the order of the number of MYB was B < W, P < B, and P < W. However, bHLH transcription factors expressions were mostly downregulated in the other two groups and presented a greater upregulation in BvsW. The bHLH transcription factor was mostly down-regulated in both groups, except for BvsW where it was further up-regulated. The bHLH transcription factor was less in purple wheat than in blue and white wheat. WRKY TFs were more downregulated in the three groups, and the order of the number of TFs was P < B, P < W, and W < B. Of the bHLH transcription factors associated with the present study color, most of the descriptions of transcription factors in the three groups are protein-coded. only gene:TraesCS2B02G289900 in BvsW is bHLH27, and the rest are protein-coded. PvsB in gene:TraesCS3D02G434900 is ICE41, gene:TraesCS5B02G422000 is phytochrome-interacting factors, transcription factor bHLH27 has 2, gene: TraesCS1A02G102400 is transcription factor TT8. There are 1 helix-loop-helix DNA-binding domain containing protein, 1 ICE41, 2 phytochrome-interacting factor, and 1 transcription factor TT8 in PvsW. A total of 19,877 new gene functions were annotated. For each differential grouping, rMATS was used to analyze the types of variable splicing events, calculate their numbers, and subsequently estimate the expression of each type of variable splicing event separately. Finally, differential analysis of AS events by the JC and JCEC quantification methods revealed that exon skipping (ES) had the largest number. The results of the RT-qPCR assay showed that the expression pattern correlated well with the sequencing results, indicating that the transcriptome sequencing results were reliable (Supplementary Figure 8).

### 3.5. Combined transcriptome and metabolome analysis

The KEGG pathway map shows that three groups of wheat were jointly enriched in the biosynthesis of amino acids, carbon metabolism, glyoxylate and dicarboxylate metabolism, amino sugar, and nucleotide sugar metabolism, starch and sucrose metabolism, and alanine, aspartate, and glutamate metabolism. Based on the results of differential metabolites and differential gene enrichment analysis, the anthocyanin biosynthesis pathway was significant in BvsW group (Supplementary Figures 9A–C). Nine quadrant plots of gene and metabolite correlations based on correlations greater than 0.8 demonstrated that many genes were consistent with differential metabolite expression patterns, with upregulation of genes and unchanged or downregulated metabolites (Supplementary Figures 9D–F). Correlation analysis was performed for differential genes and metabolites, and Pearson correlation coefficients greater than 0.8 were selected. The results of the three groups of metabolite clustering heat map showed that flavonoids and phenolic acids accounted for a major proportion of metabolites (Supplementary Figures 9G–I). The correlation network plot for BvsW, PvsB, and PvsW showed that the metabolites 1-*O-*sinapoyl-d-glucose, epigallocatechin, and catechin found in the pathways related to color synthesis (biosynthesis of phenylpropanoids, biosynthesis of flavonoids) were correlated with the genes found in the wheat lines. 1-*O-*sinapoyl-d-glucose was highly associated with seven genes in PvsW, epigallocatechin, and catechin had strong correlations with the genes in BvsW (Supplementary Figures 10A, B). The O2PLS model load plot for differential genes and metabolites revealed that the transcriptome was weighted more heavily, implying that changes in this variable perturbed the metabolome more drastically. Peonidin-3-*O-*rutinoside-5-*O-*glucoside was the metabolite that had a greater impact on the transcriptome (Supplementary Figures 10C, D).

**FIGURE 5:**
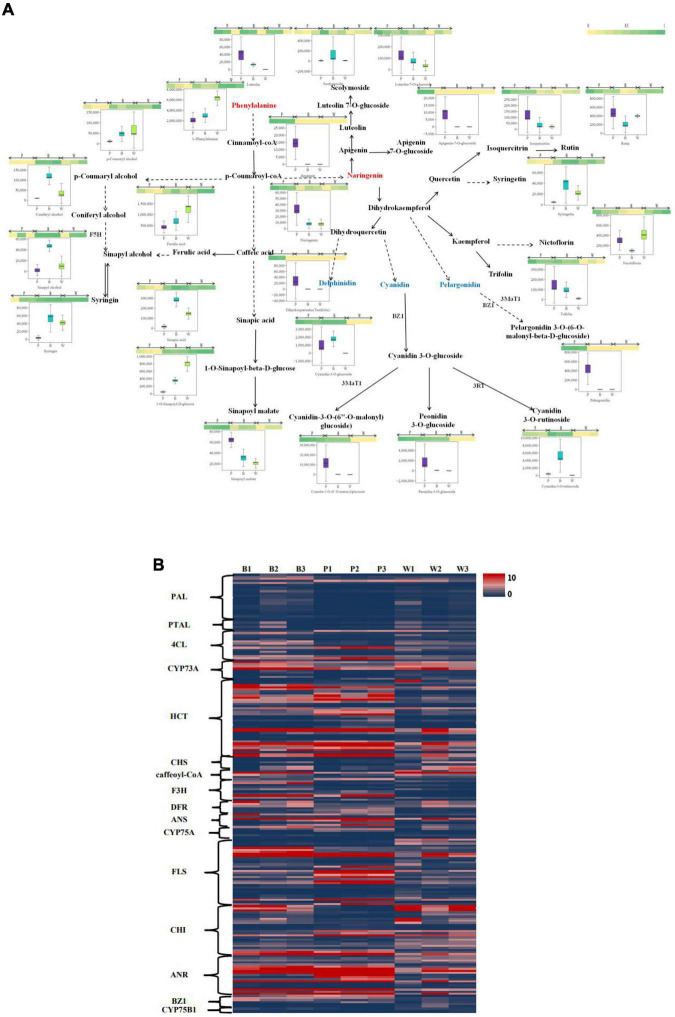
**(A)** Heat map of metabolites of the wheat seed color synthesis pathway. The pathway was constructed based on the KEGG pathway and the scientific literature. Each colored box indicates the normalized intensity of each complex ion. Box-and-whisker plots show the variation of color formation-related metabolites in each wheat variety, with the upper and lower ends representing the maximum and minimum values of metabolites, respectively (Three biological replicates × three lines, *n* = 9). **(B)** Heat map of gene expression levels (FPKM values) of key enzyme sites of the color formation pathway. PAL, phenylalanine ammonia lyase; PTAL, phenylalanine/tyrosine ammonia-lyase; 4CL, 4-coumarate-CoA ligase; CYP73A, *trans*-cinnamate 4-monooxygenase; HCT, shikimate O-hydroxycinnamoyltransferase; CHS, chalcone synthase; F3H, naringenin 3-dioxygenase; DFR, bifunctional dihydroflavonol 4-reductase/flavanone 4-reductase; ANS, anthocyanidin synthase; CYP75A, flavonoid 3′,5′-hydroxylase; FLS: flavonol synthase; CHI, chalcone isomerase; ANR, anthocyanidin reductase; CYP75B1, flavonoid 3′-monooxygenase; BZ1, anthocyanidin 3-O-glucosyltransferase.

An integrative analysis of upregulated and downregulated differential metabolites in BvsW, PvsB, and PvsW related to color formation pathways (i.e., biosynthesis of phenylpropanoids, biosynthetic of flavonoids, anthocyanin, isoflavone, and flavonoid and flavonol, Figure 5A) revealed 1 L-phenylalanine, 9 phenolic acid analogs, and 17 flavonoids. L-phenylalanine, ferulic acid, nicotiflorin, 1-*O-*sinapoyl-d-glucose were higher in the W group. Naringenin, dihydroquercetin, cyanidin-3-*O-*(6″-*O-*malonyl) glucoside, peonidin-3-*O-*glucoside, and pelargonidin were higher in P. Cyanidin-3-*O-*glucoside, cyanidin-3-*O-*rutinoside, and coniferyl alcohol were higher in B. The clustering analysis of FPKM (Figure 5B), the upregulated and downregulated genes of key enzyme sites in the color formation pathway, showed that the expression of structural genes of key enzyme sites in the color formation pathway was higher in B and P than in W, and higher in P than that in B. CYP73A, HCT, F3H, DFR, FLS, and ANR enzymes were more prominent. Among all the genes upregulated and downregulated at key enzyme sites in the color-associated pathway, the gene TraesCS2D02G392900 in naringenin 3-dioxygenase (F3H) was found to be relatively highly expressed in colored wheat but not in white wheat.

## 4. Discussion

The purpose of this study was to investigate the causes of grain color formation in three different types of wheat at maturity. The concentration of anthocyanin usually increases when exposed to adverse environmental factors (30), and it generally accumulates in the nutritional and reproductive parts of the plants to protect them from excessive ultraviolet radiation. The environment conditions of this study were made consistent for all the studied wheat lines. This was done to ensure that the observed phenotypic patterns are the result of differences in biomolecular architecture rather than an expression of phenotypic plasticity in response to changing environmental conditions. For example, previous studies have shown that *Ba, Pp*, and *Psy* genes are the main reasons for a blue aleurone, purple pericarp, and yellow endosperm, respectively (8). In addition to the genetic structure, grain color is associated with the presence and composition of anthocyanins. Blue grains differ from purple grains in the composition and presence of individual anthocyanins (31). Regarding anthocyanin content, previous studies have stated that the darker the color of wheat, the higher is the anthocyanin content, showing a trend of deep/dark purple grain > blue grain > purple grain > red grain > amber grain wheat (20). It has also been shown that crossing genetically distinct lines (e.g., one with a purple pericarp and one with a blue aleurone) can increase the anthocyanin content (32). Nine anthocyanins were detected in this study, out of which cyanidin, peonidin, and pelargonidin were dominant. There were 8, 7, and 1 anthocyanins in purple grain, blue grain, and white-grain, respectively. It was observed that purple wheat had the highest anthocyanin concentration and white wheat had the lowest among the three different grain colors of wheat. The results of the previous study that the darker the color, the higher is the anthocyanin content were again verified. Although colored wheat has a higher anthocyanin content than regular wheat, it is generally consumed at high temperatures, however, heat processing reduces the total phenolic content and antioxidant activity. Studies have shown that fresh grains have a higher total phenolic content than mature grains. These results suggest that colored wheat fresh grains should be used for the production of nutritious foods (33).

Regarding anthocyanin composition, many studies have identified delphinidin as the major anthocyanin in blue aleurone wheat, whereas cyanidin-3-glucoside is the main anthocyanin of purple pericarp (31). For blue aleurone wheat, the main anthocyanins were delphinidin 3-glucoside and delphinidin 3-rutinoside, while unlike in the purple grains, cyanidin 3-glucoside and cyanidin 3-rutinoside were present in smaller amounts (34). Cyanidin-3-glycoside is the major anthocyanin, not only in blue-grain wheat but also in purple-grain wheat (31). Additionally, peonidin-3-*O-*glucoside, malvidin-3-*O-*galactoside, and cyanidin-3-*O-*glucoside are the significant anthocyanins in purple wheat (22). Studies have shown that purple wheat is mainly composed of five anthocyanins, namely, cyanidin-3-glucoside, cyanidin-3-(6-malonyl glucoside), cyanidin-3-rutinoside, peonidin-3-glucoside, and peonidin-3-(6-malonylglucoside) (35). Acylated anthocyanins containing malonyl and succinyl groups exist only in purple wheat (36). Hosseinian et al. (37) identified 13 anthocyanins in purple wheat, among which the cyanidin-3-glucoside content was the highest, followed by cyanidin-3-galactoside and anthocyanin-3-glucoside. Purple wheat anthocyanins exist in the form of pelargonidin-3-glycosides and arabinosylated anthocyanins. In this study, peonidin-3-*O-*(6″-*O-*acetyl) glucoside and pelargonidin-3-*O-*(6″-*O-*malonyl) glucoside are only present in purple-grain wheat, while peonidin-3-*O-*rutinoside-5-*O-*glucoside is present only in blue-grain wheat. The highest contents of cyanidin-3-*O-*(6″-*O-*malonyl) glucoside and cyanidin-3-*O-*rutinoside (keracyanin) were found in purple- and blue-grain wheat, respectively. White wheat contained only keracyanin, which differed from the results of previous studies, probably because each wheat variety has a specific anthocyanin profile. Although many studies have identified feijoa glycosides as the major anthocyanins in blue paste wheat (31), this finding was not confirmed in this study. This may be due to the transferred chromosomal segments of *Thinopyrum ponticum* in blue-grained wheats having a different length and position, resulting in great genetic diversity among individual blue grain genomes (8). The various studies above confirm previous findings or find contradictory results, which may be due in part to different genotypes and growing sites resulting in unique chemical characteristics and specific compounds for each anthocyanin-rich strain (38).

At the metabolic level, color synthesis begins with L-phenylalanine of phenylpropane biosynthesis, which is regulated by various genes. Naringenin as a color turning point, starts the flavonoid biosynthesis pathway, further affecting the anthocyanin biosynthesis, flavonoid and flavonol biosynthesis. Naringenin forms dihydrokaempferol under the regulation of F3H. Cyanidin and pelargonidin are formed under the regulation of bifunctional dihydroflavonol 4-reductase (DFR). In this experiment, purple wheat contained more naringenin, which may lead to the highest amount of anthocyanin precursor dihydroquercetin in purple wheat in the anthocyanin biosynthesis pathway. This would result in the production of many kinds of anthocyanins at high concentrations, resulting in darker grain color. In addition to the influence of metabolites in flavonoid and anthocyanin biosynthesis pathways, color formation is also affected by structural genes and transcriptional factors. In the KEGG color synthesis pathway, the expression of structural genes in colored wheat is generally higher than that in white wheat, with mainly F3H and bifunctional DFR genes regulating the formation of cyanidin and pelargonidin. In particular, the relative expression of TraesCS2D02G392900 gene in F3H was relatively high in colored wheat, whereas this gene was not detected in white wheat. The biosynthesis of anthocyanin were mainly affected by MYB TFs, bHLH, WD40 protein, and WRKY, among which bHLH TFs and WD40 proteins acted as a bridge to promote anthocyanin biosynthesis (39–41). In this experiment, it was found that bHLH TFs were typically downregulated in PvsB and PvsW and upregulated in BvsW. In other words, the expression of bHLH TFs in purple wheat was higher than that in blue wheat and white wheat, which promoted anthocyanin biosynthesis. In summary, in the presence of various substrates and enzymes, the anthocyanin content of purple wheat is higher than those of blue wheat and white wheat, which may be the reason for the higher anthocyanin content in darker wheat grains.

## 5. Conclusion

Metabolomic analyses revealed 628 metabolites, including 102 types of flavonoids and nine kinds of anthocyanins related to color formation, mainly cyanidin, and peonidin. The most abundant species and content of anthocyanins was observed in purple wheat, followed by blue wheat and white wheat. Peonidin-3-*O-*(6″-*O-*acetyl) glucoside and pelargonidin-3-*O-*(6″-*O-*malonyl) glucoside are only present in purple-grain wheat, while peonidin-3-*O-*rutinoside-5-*O-*glucoside is only present in blue-grain wheat. Similarly, cyanidin-3-*O-*rutinoside (keracyanin) is the only anthocyanin present in white wheat. The expression of the structural gene F3H and bifunctional DFR, which controls color formation, was higher in colored wheat than in white wheat. The expression of the gene TraesCS2D02G392900 in F3H was higher in colored wheat grains but not detected in white wheat, indicating its importance in the final grain color formation. The expression of bHLH transcription factor was high in purple wheat. In summary, the wheat color is mainly influenced by flavonoids from the metabolome; however, the biosynthetic pathway of flavonoid metabolites is influenced by structural genes and TFs related to color formation at the transcriptional level, thus, elucidating the causes of wheat seed color formation.

## Data availability statement

The data presented in this study are deposited in the NCBI repository, accession number: PRJNA858194.

## Author contributions

LL: writing—original draft and methodology. HZ: conceptualization and writing—review and editing. JL: formal analysis and methodology. TH: data curation and visualization. XZ and HX: data curation and investigation. YG and QW: methodology and visualization. PZ: formal analysis and investigation. PQ: supervision, project administration, and funding acquisition. All authors contributed to the article and approved the submitted version.

## Footnotes

1
http://www.anpel.com.cn/

2
www.shimadzu.com.cn/

3
http://www.metware.cn/
