# Supplementary material for: Grain color formation and analysis of correlated genes by metabolome and transcriptome in different wheat lines at maturity
Source: Front Nutr. 2023 Feb 7;10:1112497. doi: 10.3389/fnut.2023.1112497 (PMC9941320; doi:10.3389/fnut.2023.1112497)
Supplement: Supplementary file 1 [file Data_Sheet_1.zip › supplementary materials/Supplementary Figure.docx]

| A(N) | B(P) |
| --- | --- |
| 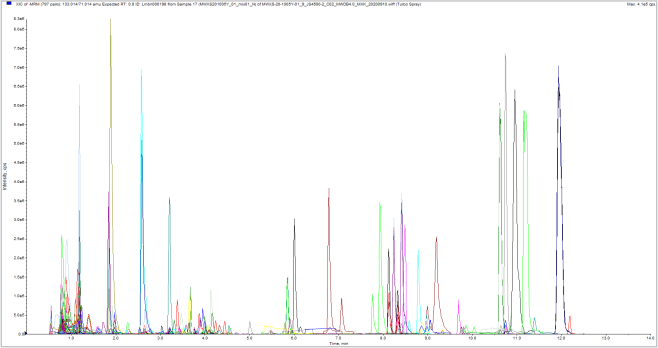 | 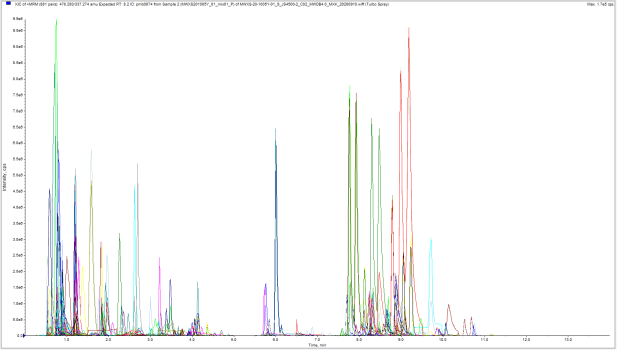 |
| C(N) | D(P) |
| 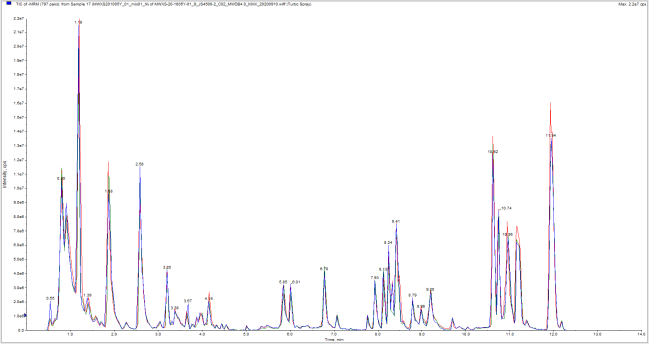 | 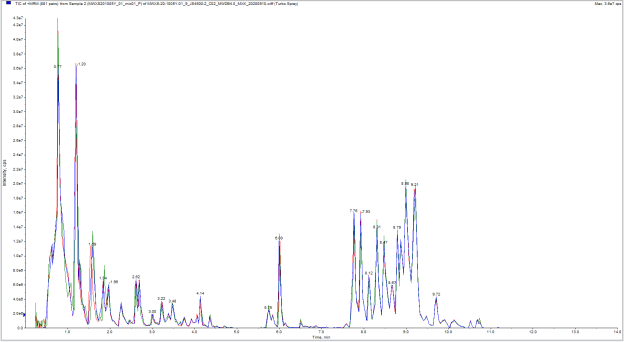 |

**Fig. S1.** (A-B) MRM metabolite detection multi-peaks; (C-D) QC sample mass spectrometry detection TIC overlay; N stands for negative ion mode and P stands for positive ion mode.

| A (BvsW) | B (PvsB) | C (PvsW) |
| --- | --- | --- |
| 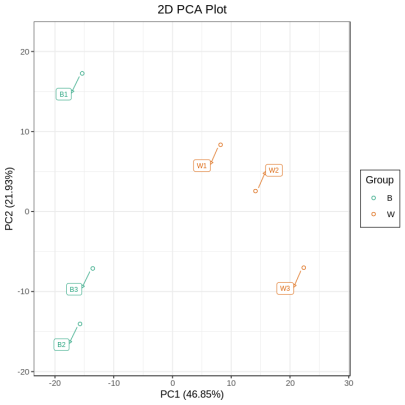 | 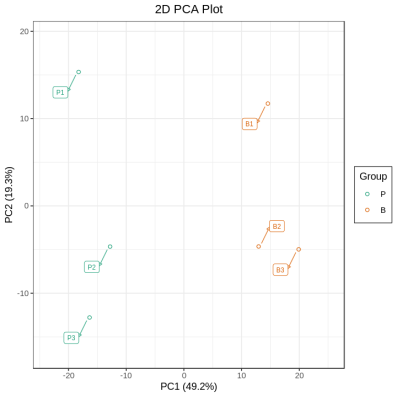 | 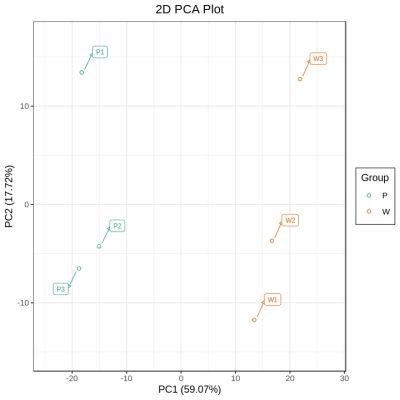 |
| D (BvsW) | E (PvsB) | F (PvsW) |
| 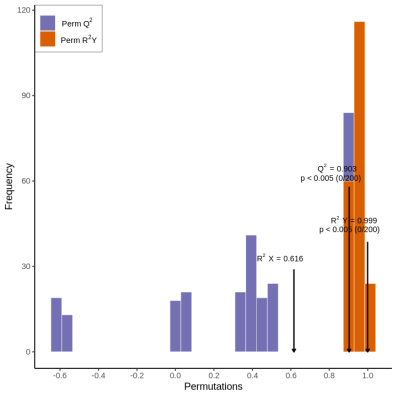 | 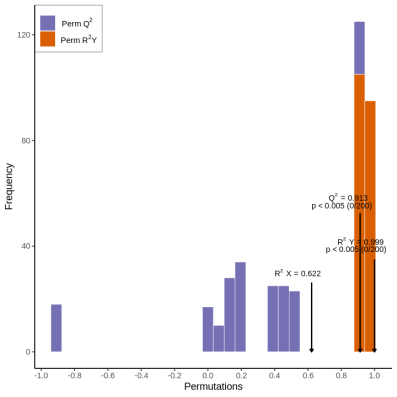 | 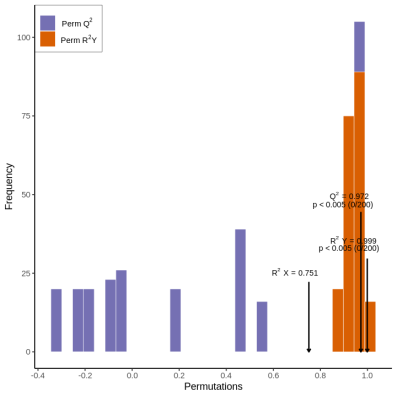 |
| G (BvsW) | H (PvsB) | I (PvsW) |
| **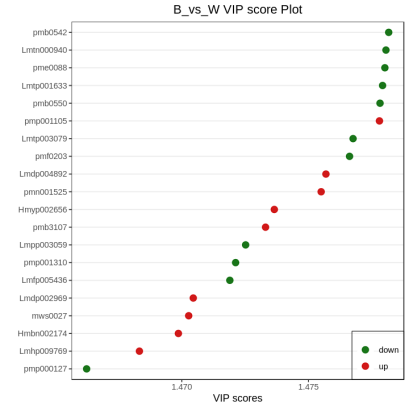** | **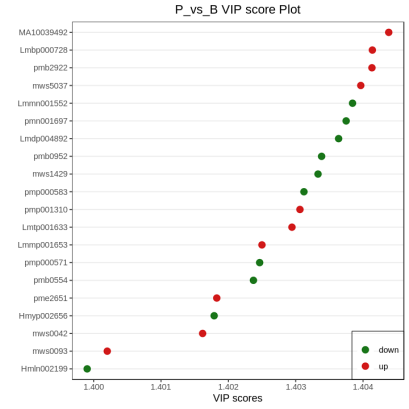** | **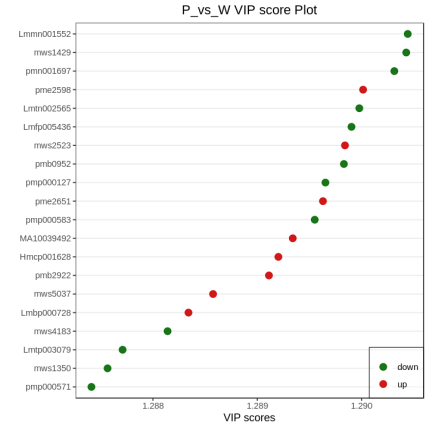** |

**Fig. S2.** (A-C) Grouped principal component analysis plots. pc1 denotes the first principal component, pc2 denotes the second principal component; (D-F) OPLS-DA validation plots; G-I) differential metabolite VIP value plots. R2X and R2Y indicate the explanation rate of the proposed model for X and Y matrices, respectively, and Q2 indicates the predictive ability of the model, and the closer these three indicators are to 1, the more stable and reliable the model is.

| A (PvsB__BvsW) | B (PvsB__PvsW) | C (PvsW__BvsW) |
| --- | --- | --- |
| 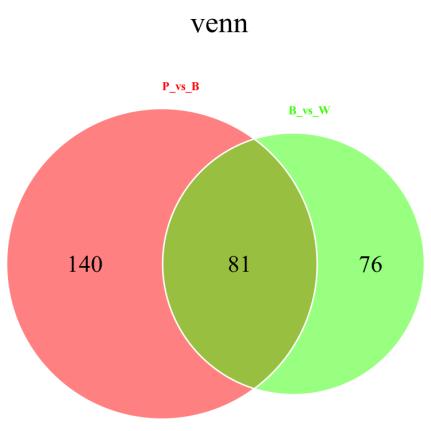 | 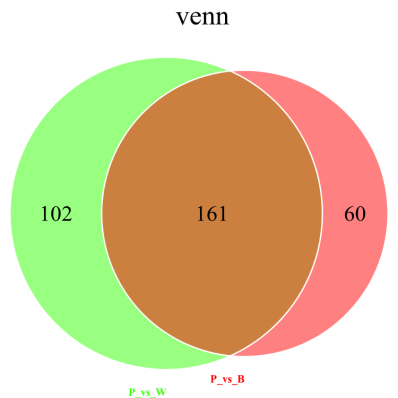 | 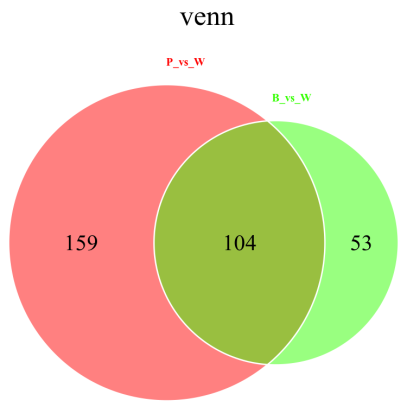 |
| D (PvsB__PvsW__BvsW) | | |
| 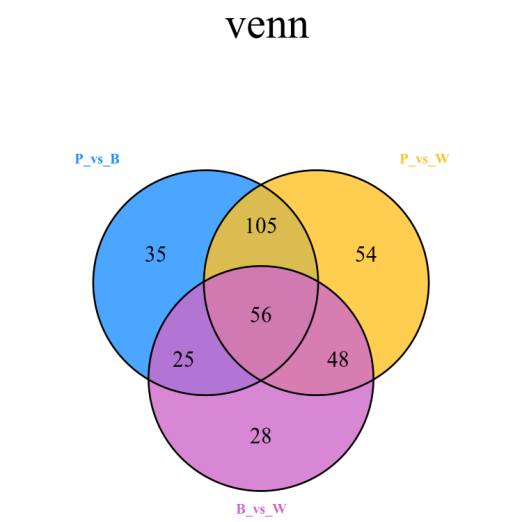 | | |

**Fig. S3.** Venn diagram of differences among groups.

| A (BvsW) | B (PvsB) | C (PvsW) |
| --- | --- | --- |
| 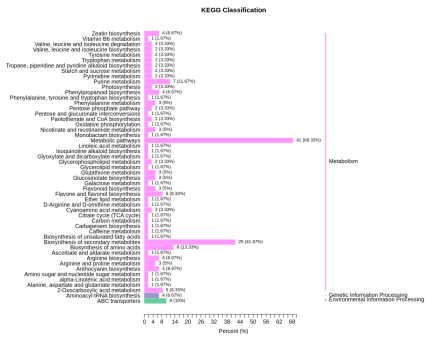 | 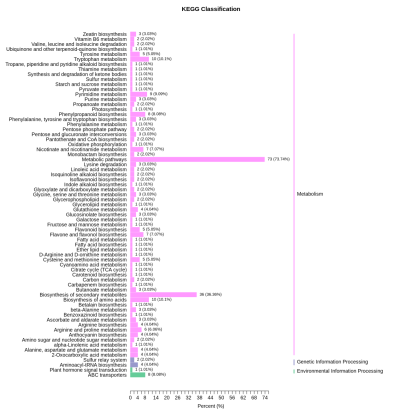 | 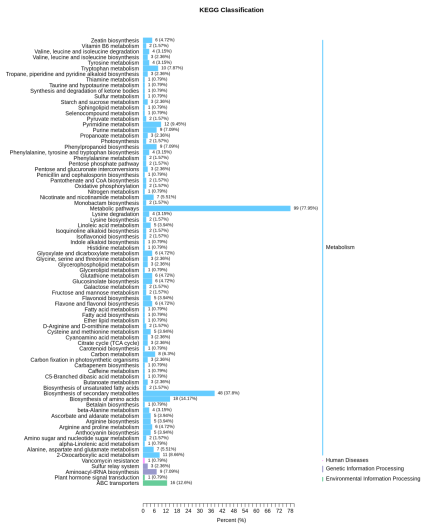 |
| D (BvsW) | E (PvsB) | F (PvsW) |
| 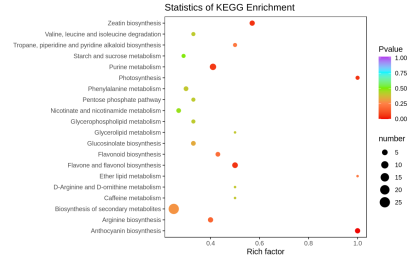 | 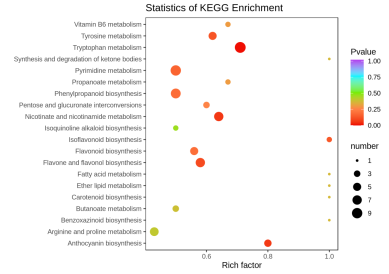 | 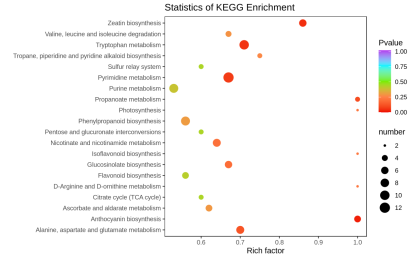 |

**Fig. S4.** (A-C) Differential metabolite KEGG classification maps. The vertical coordinate is the name of the KEGG metabolic pathway, the horizontal coordinate is the number of metabolites annotated to the pathway and its ratio to the total number of metabolites annotated; (D-F) Differential metabolite KEGG enrichment map . The horizontal coordinate indicates the Rich factor of each pathway, the vertical coordinate is the name of the pathway, and the color of the dot is the Pvalue, the more red means the more significant enrichment. The size of the dots represents the number of enriched metabolites.

| A | | B | | |
| --- | --- | --- | --- | --- |
| 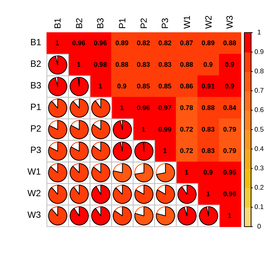 | | | 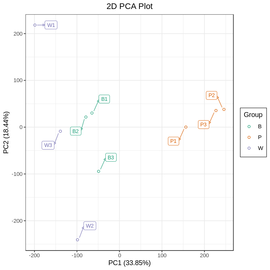 | |
| C (BvsW) | D (PvsB) | | | E (PvsW) |
| 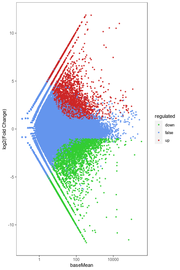 | 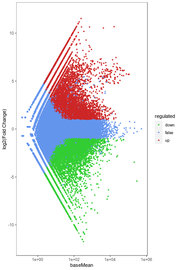 | | | 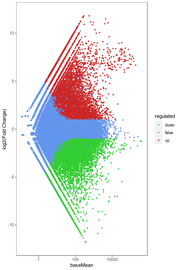 |
| F | | | | |
| 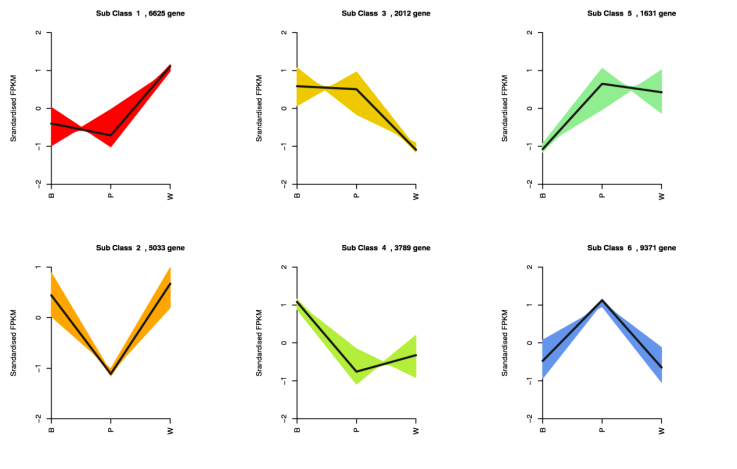 | | | | |

**Fig. S5.** (A) Correlation heat map;(B) PCA map; (C-E) MA map of differential genes; (F) Kmeans clustering map of gene expression

| A(BvsW) | B(PvsB) | | | C(PvsW) |
| --- | --- | --- | --- | --- |
| 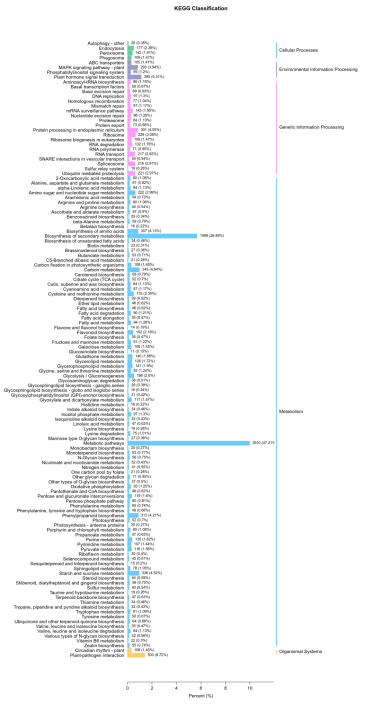 | 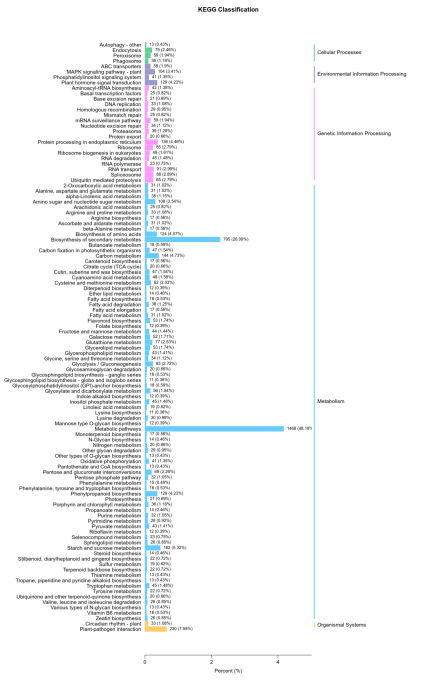 | | | 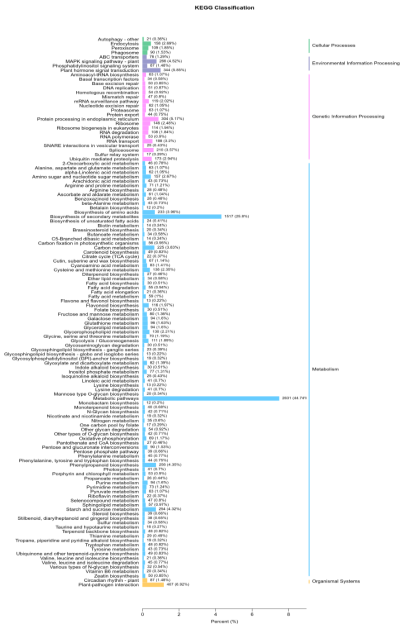 |
| D(BvsW) | | | E(PvsB) | |
| 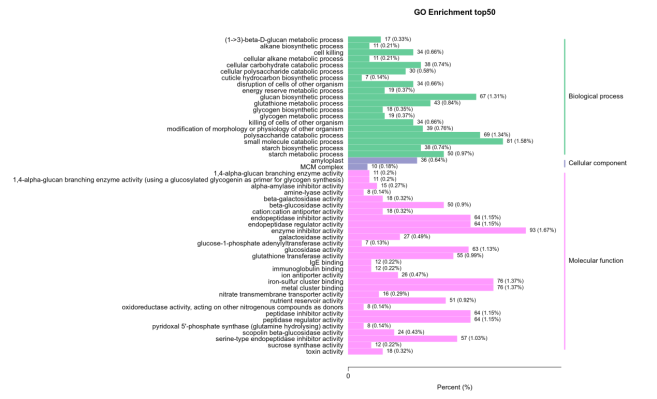 | | 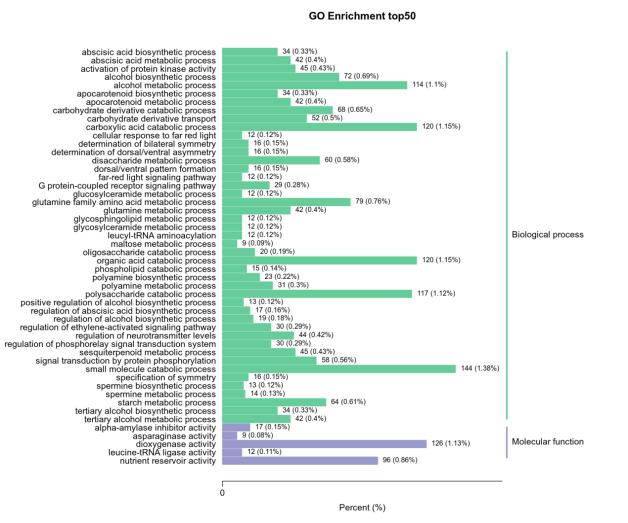 | | |
| F(PvsW) | | | | |
| 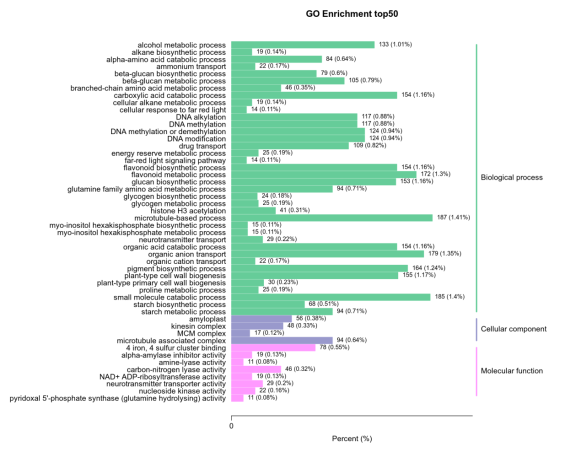 | | | | |

**Fig. S6.** (A-C) KEGG classification bars of differentially expressed genes; (D-E) GO enrichment bars of differentially expressed genes

| A (BvsW) |
| --- |
| 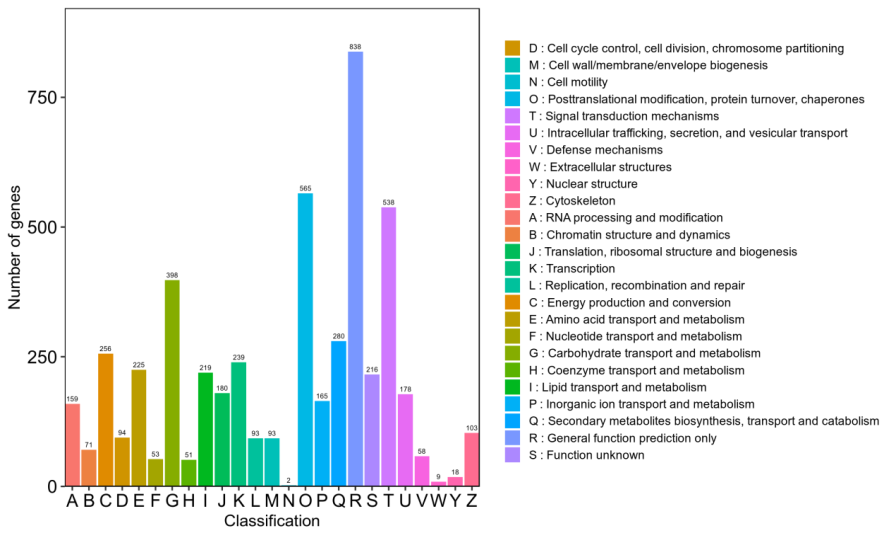 |
| B (PvsB) |
| 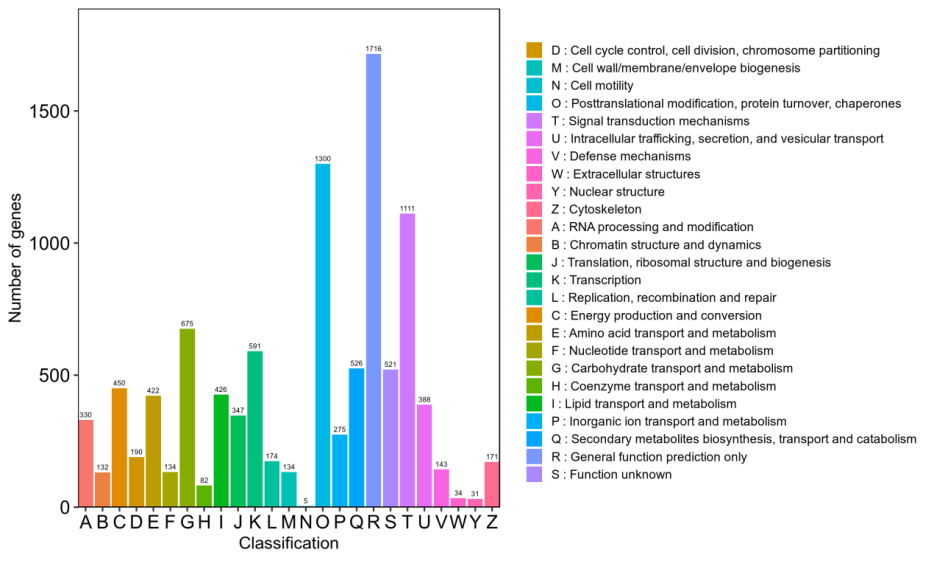 |
| C (PvsW) |
| 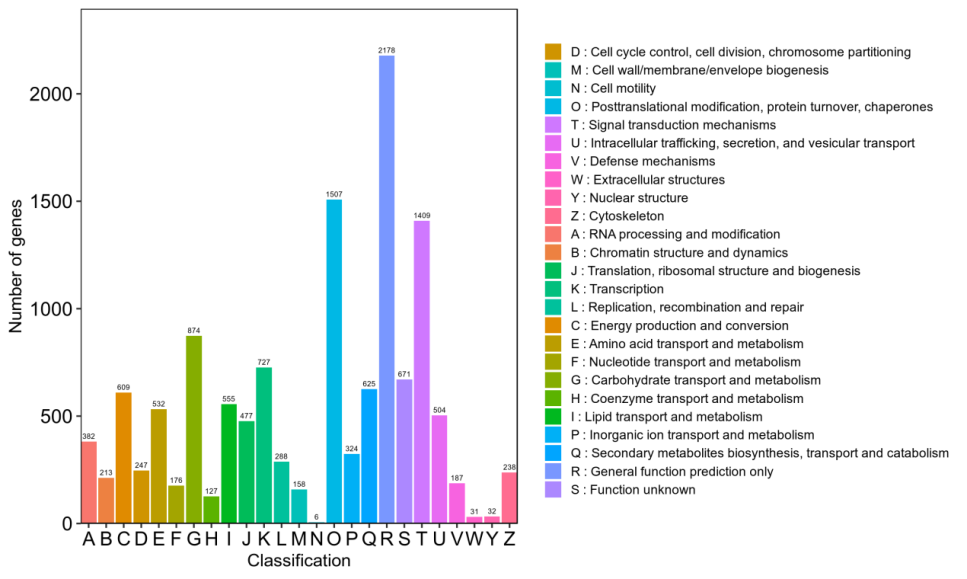 |

**Fig. S7.** (A-C) KOG classification bar chart

| 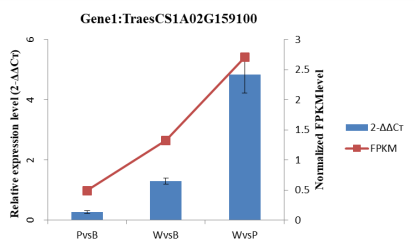 | 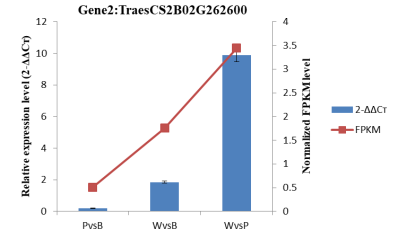 | 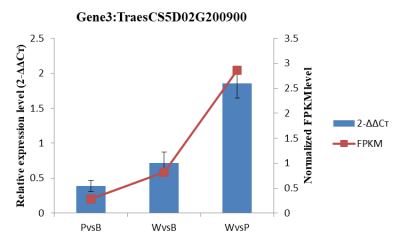 |
| --- | --- | --- |
| 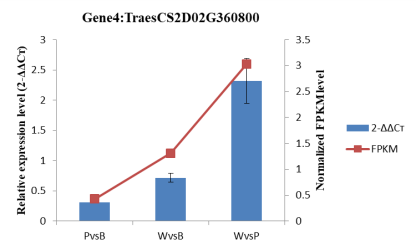 | 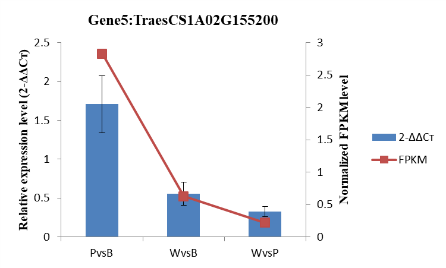 | 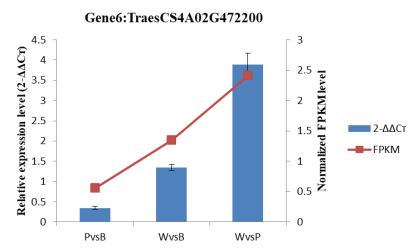 |
| 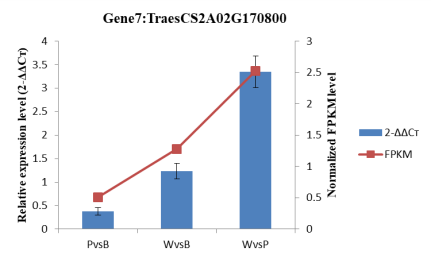 | 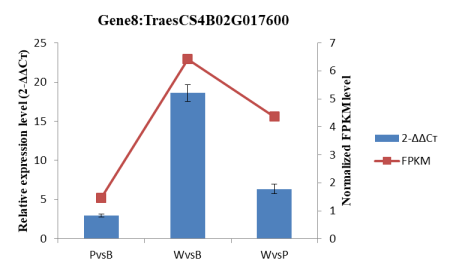 | 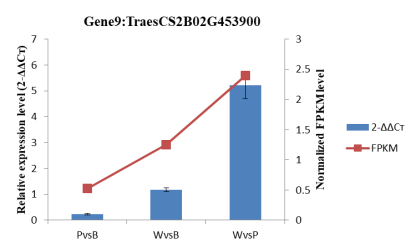 |

**Fig. S8.** qRT-PCR validation of transcription levels of randomly selected DEGs.

| A(BvsW) | B(PvsB) | C(PvsW) |
| --- | --- | --- |
| 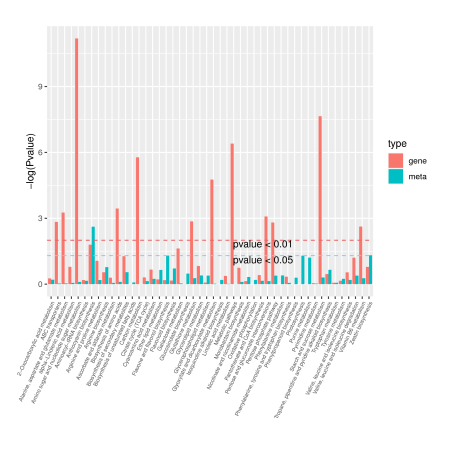 | 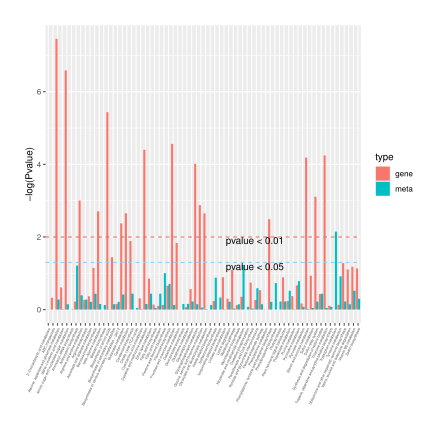 | 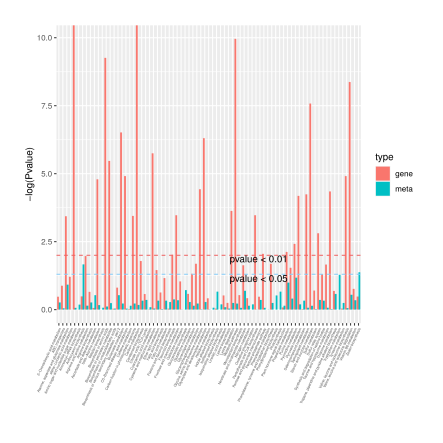 |
| D(BvsW) | E(PvsB) | F(PvsW) |
| 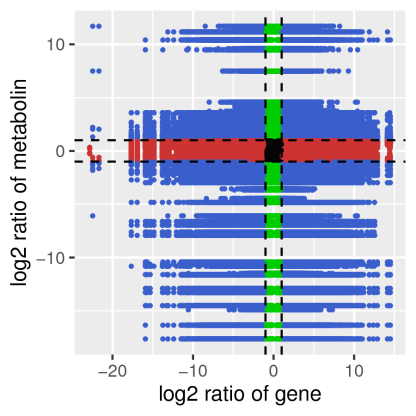 | 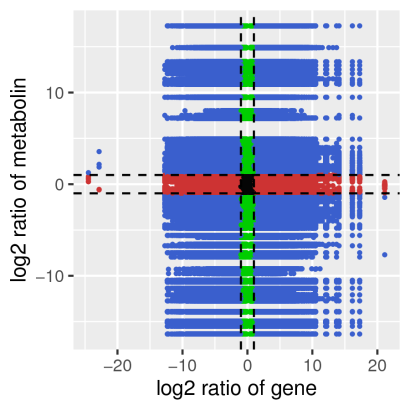 | 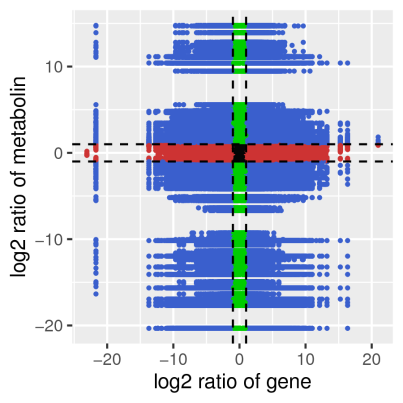 |
| G(BvsW) | H(PvsB) | I(PvsW) |
| 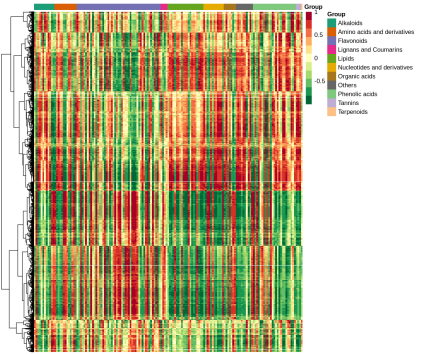 | 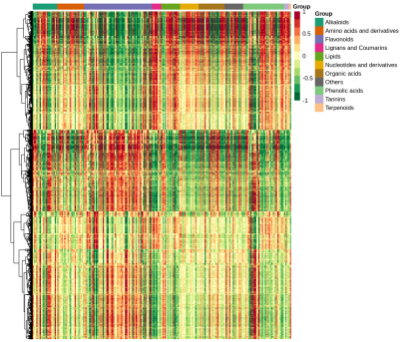 | 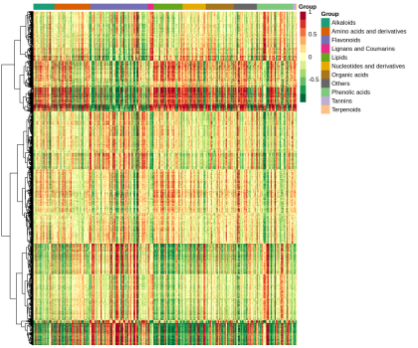 |

**Fig. S9.** (A-C) KEGG enrichment analysis pvalue histogram; (D-F) Correlation analysis nine-quadrant plot; (G-I) Correlation coefficient clustering heat map

| A（left-right:：BvsW、PvsB、PvsW） | | | | |
| --- | --- | --- | --- | --- |
| 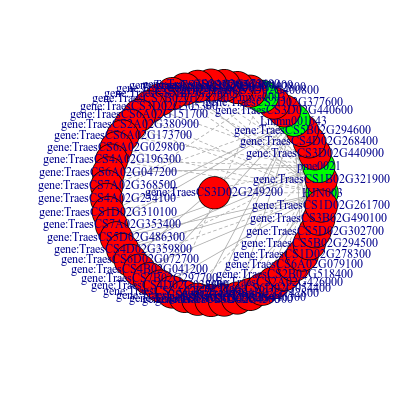 | 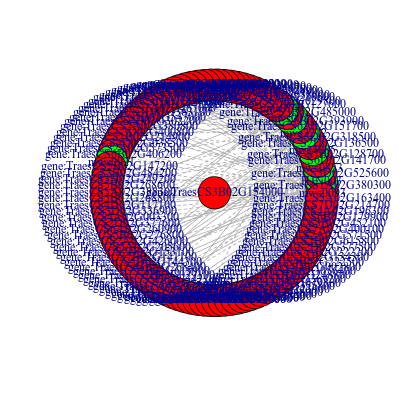 | | | 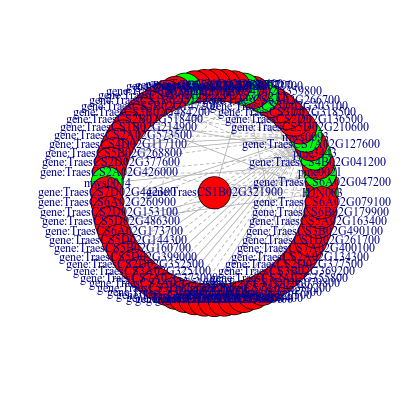 |
| B（left-right:：BvsW、PvsB、PvsW） | | | | |
| 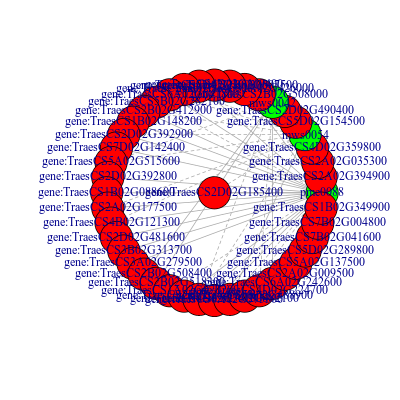 | 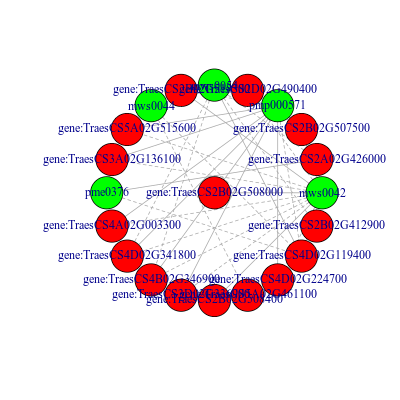 | | | 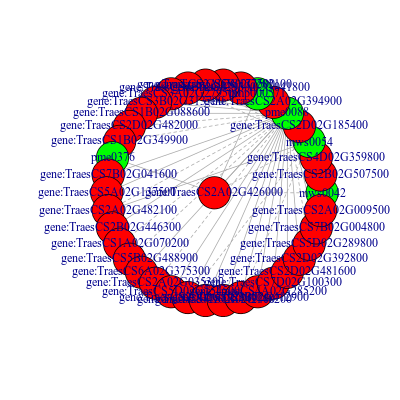 |
| C | | D | | |
| 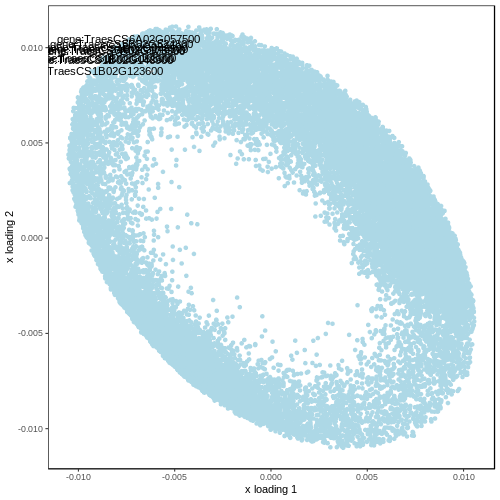 | | | 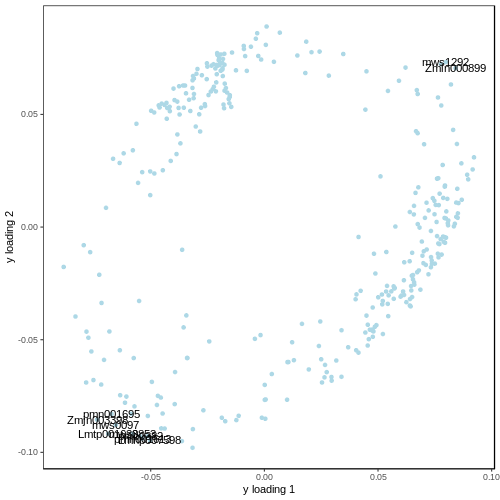 | |

**Fig. S10.** (A-B) Typical correlation analysis plots. Metabolites are marked in green and genes are marked in red, solid lines represent positive correlations and dashed lines represent negative correlations; (C-D) O2PLS model loading plot. The distance of each point to the origin means the magnitude of correlation with another histology, and the top 10 genes and metabolites that have a large impact on another histology are marked in the figure.
