# Supplementary material for: Grain color formation and analysis of correlated genes by metabolome and transcriptome in different wheat lines at maturity
Source: Front Nutr. 2023 Feb 7;10:1112497. doi: 10.3389/fnut.2023.1112497 (PMC9941320; doi:10.3389/fnut.2023.1112497)
Supplement: Supplementary file 1 [file Data_Sheet_1.zip › supplementary materials/Supplementary Table.docx]

**Supplementary Table**

**Table 1**

Gene primer sequences in qRT-PCR

| **Gene ID** | **Forward /Reverse primer** | **Primer sequence (5′to 3′)** |
| --- | --- | --- |
| gene:TraesCS1A02G159100 | Forward primer | CAAGACCGTGAAGAAGAC |
| gene:TraesCS1A02G159100 | Reverse primer | CTTGTTGGTGTGGAAGTC |
| gene:TraesCS2B02G262600 | Forward primer | TATGTGGTCTACCGTATC |
| gene:TraesCS2B02G262600 | Reverse primer | CAGTAGGAGTTGAGTACA |
| gene:TraesCS5D02G200900 | Forward primer | TTTATCCGAGAAGCCTCT |
| gene:TraesCS5D02G200900 | Reverse primer | CTGGATGTAGTGCATCTG |
| gene:TraesCS2D02G360800 | Forward primer | GAGGACCAACAGGACTTT |
| gene:TraesCS2D02G360800 | Reverse primer | CCTTGAATTGCTCCGATAT |
| gene:TraesCS1A02G155200 | Forward primer | AAGGAAGGATGTTATGAC |
| gene:TraesCS1A02G155200 | Reverse primer | ACAACTGATGTTCTTCTC |
| gene:TraesCS4A02G472200 | Forward primer | GAGAAGAAGGTGAGGAAG |
| gene:TraesCS4A02G472200 | Reverse primer | GCATTTCTGGTTGATGAG |
| gene:TraesCS2A02G170800 | Forward primer | TCTTCGACAAGTACCATC |
| gene:TraesCS2A02G170800 | Reverse primer | GTAGAACCTGTTGCTGAG |
| gene:CS4B02G017600 | Forward primer | GATTCTCCACACCATCTC |
| gene:CS4B02G017600 | Reverse primer | CATCCGAGGTCTTCAAAG |
| gene:CS2B02G453900 | Forward primer | CGTGGTATCAATGCTATG |
| gene:CS2B02G453900 | Reverse primer | CTGACACTCTTGAGGTTA |
| 26S(Reference gene) | Forward primer | GCTGGCTCGTTCAACTGATG |
| 26S(Reference gene) | Reverse primer | GGACCAAGCGTTCTGATTACTC |

**Table 2**

Statistics of the number of differential metabolites

| **Group name** | **All sig diff** | **Up-regulated** | **Down-regulated** |
| --- | --- | --- | --- |
| B vs W | 157 | 65 | 92 |
| P vs B | 221 | 105 | 116 |
| P vs W | 263 | 108 | 155 |

**Table 3**

351 flavonoids classification

| **Class** | **Number** |
| --- | --- |
| Amino acids and derivatives | 43 |
| Phenolic acids | 53 |
| Nucleotides and derivatives | 26 |
| Flavonoids | 83 |
| Lignans and Coumarins | 10 |
| Others | 27 |
| Tannins | 4 |
| Alkaloids | 27 |
| Terpenoids | 2 |
| Organic acids | 33 |
| Lipids | 43 |
| Total | 351 |

**Table 4**

Overview of transcriptome sequencing datasets and quality checks

| **Sample** | **Raw Reads** | **Clean Reads** | **Clean Base(G)** | **Error Rate(%)** | **Q20(%)** | **Q30(%)** | **GC Content(%)** |
| --- | --- | --- | --- | --- | --- | --- | --- |
| B1 | 46648628 | 44116048 | 6.62 | 0.03 | 97.58 | 93.76 | 55.83 |
| B2 | 43487908 | 41172430 | 6.18 | 0.03 | 97.22 | 92.78 | 55.48 |
| B3 | 54989192 | 52620614 | 7.89 | 0.02 | 98.04 | 94.43 | 56.71 |
| P1 | 45540010 | 43732846 | 6.56 | 0.02 | 97.99 | 94.31 | 58.82 |
| P2 | 54600308 | 51649476 | 7.75 | 0.02 | 97.99 | 94.29 | 58.97 |
| P3 | 45501254 | 43588240 | 6.54 | 0.02 | 98.21 | 94.84 | 59.29 |
| W1 | 47694222 | 45219122 | 6.78 | 0.03 | 96.9 | 92.21 | 52.38 |
| W2 | 49524420 | 47864806 | 7.18 | 0.03 | 97.85 | 93.91 | 56.15 |
| W3 | 45189628 | 43002260 | 6.45 | 0.03 | 97.49 | 93.34 | 53.57 |

**Table 5**

Up- and down-regulation statistics of differentially expressed genes

| **Group name** | **All DEGs** | **Up-regulated** | **Down-regulated** |
| --- | --- | --- | --- |
| B vs W | 8357 | 3957 | 4400 |
| P vs B | 17427 | 8316 | 9111 |
| P vs W | 21703 | 9762 | 11941 |
